# The Arabidopsis *AtRaptor *genes are essential for post-embryonic plant growth

**DOI:** 10.1186/1741-7007-3-12

**Published:** 2005-04-21

**Authors:** Garrett H Anderson, Bruce Veit, Maureen R Hanson

**Affiliations:** 1 Molecular Biology and Genetics, Cornell University, Ithaca, 14853, USA; 2 AgResearch, Private Bag 11008, Tennent Drive, Palmerston North, 5301, New Zealand

## Abstract

**Background:**

Flowering plant development is wholly reliant on growth from meristems, which contain totipotent cells that give rise to all post-embryonic organs in the plant. Plants are uniquely able to alter their development throughout their lifespan through the generation of new organs in response to external signals. To identify genes that regulate meristem-based growth, we considered homologues of Raptor proteins, which regulate cell growth in response to nutrients in yeast and metazoans as part of a signaling complex with the target of rapamycin (TOR) kinase.

**Results:**

We identified *AtRaptor1A *and *AtRaptor1B*, two loci predicted to encode Raptor proteins in Arabidopsis. Disruption of *AtRaptor1B *yields plants with a wide range of developmental defects: roots are thick and grow slowly, leaf initiation and bolting are delayed and the shoot inflorescence shows reduced apical dominance. *AtRaptor1A AtRaptor1B *double mutants show normal embryonic development but are unable to maintain post-embryonic meristem-driven growth. *AtRaptor *transcripts accumulate in dividing and expanding cells and tissues.

**Conclusion:**

The data implicate the TOR signaling pathway, a major regulator of cell growth in yeast and metazoans, in the maintenance of growth from the shoot apical meristem in plants. These results provide insights into the ways in which TOR/Raptor signaling has been adapted to regulate plant growth and development, and indicate that in plants, as in other eukaryotes, there is some Raptor-independent TOR activity.

## Background

Plant development is remarkably plastic. Groups of totipotent cells termed meristems, which are maintained throughout the life of the plant, give rise to all post-embryonic organs from roots and leaves to petals and fruit. This allows plants, unlike metazoans, to change their final body plans dramatically in response to environmental, hormonal and nutritional cues. While much has been learned about the determination of cell fates in the embryo [1] and the apical meristems [2-4], less is known about the genes that control the growth that occurs in cells emerging from the plant meristem.

TOR proteins were originally identified in budding yeast as the targets of rapamycin, a potent antibiotic that disrupts cell growth [5,6]. In both yeast and metazoans, TOR proteins mediate translation in response to nutrients [7]. Yeast TOR2 and mammalian mTOR also regulate cytoskeletal organization [8-13].

In both yeast and mammalian cells, TOR proteins form a complex, TORC1, with GβL [14] and Raptor (regulatory associated protein of TOR) [15-17]; their yeast homologues are LST8 and KOG1, respectively [17]. Raptor contains protein-binding domains, and the Raptor N-terminus shows similarity to a caspase domain [18], though catalytic activity has yet to be shown. It has been reported that the strength of the TOR-Raptor interaction is regulated by nutrients, though there is not yet consensus on this point [15-17]. Raptor functions in TORC1 to recruit substrates for phosphorylation by TOR; Raptor binds TOR substrates S6kinase and eIF4E-BP, and is necessary for full TOR phosphorylation of these substrates *in vitro *[16,19]. The TOR-Raptor complex is thought to mediate the nutrient-sensitive regulation of cell growth; mutants in yeast lacking a functional complex cease cell growth in a manner that mimics rapamycin treatment [17]. A second TOR complex, TORC2, involves GβL, Rictor (rapamycin-insensitive companion to TOR) and perhaps other proteins [13,17,20]. This complex is unaffected by rapamycin, and is thought to mediate TOR cytoskeletal regulation.

In *Arabidopsis *the single TOR homologue, AtTOR, is critical for plant development [21,22]. *AtTOR *insertion homozygote embryos undergo cell division but are unable to gain cell volume or undergo apical-basal differentiation. Although *AtTOR *transcripts accumulate in all tissues assayed [22], an AtTOR-Gus fusion transcript is only translated in meristematic tissue and the immediately adjacent expanding cells [21]. A TOR-specific microRNA conserved between Arabidopsis and rice [23] implicates post-transcriptional regulation in *AtTOR *expression. Rapamycin perturbs the activity of the nutrient-sensitive TOR complex in yeast and mammals. Arabidopsis, however, is insensitive to rapamycin, [21] precluding this approach to the elucidation of Arabidopsis TOR signaling.

Here we report a characterization of *AtRaptor1A *and *AtRaptor1B*, which encode the two *Arabidopsis *Raptor homologues. We found defects in both root and shoot growth in *AtRaptor1B *disruption lines, resulting in slow leaf initiation, late flowering and increased branching. We also found that *AtRaptor1A AtRaptor1B *double mutant homozygotes undergo normal seedling development, but exhibit only minimal post-embryonic meristem activity. We contrast these results with those found previously for the *AtTOR *mutant, and discuss the implications of this work for TOR signaling in plants.

## Results

### Characterization of the Arabidopsis Raptor homologues

To search for plant homologues of mammalian Raptor, we surveyed the completed *Arabidopsis *genome and found two loci that might encode proteins highly similar to Raptor at the north ends of chromosomes 3 and 5. We did not find an expressed sequence tag (EST) for the first locus (*AtRaptor1A*; At5g01770). However, we used the 5'-rapid amplification of cDNA ends (RACE) to establish that the locus is transcribed to an mRNA with a 5' end consistent with the predicted open reading frame (ORF) of *AtRaptor1A*. The AtRaptor1A protein is predicted to be 1,346 amino acids in length. The second locus (*AtRaptor1B*; At3g08850) was represented by a single EST (accession no. AY769948). We used 5' RACE to confirm that the EST contained the full ORF of *AtRaptor1B*. The *AtRaptor1B *locus is transcribed to a 4.8 kb mRNA containing 23 exons, and the protein is predicted to be 1,344 amino acids in length. Predicted AtRaptor1A and AtRaptor1B proteins show 80% identity over their entirety.

**Figure 1:**
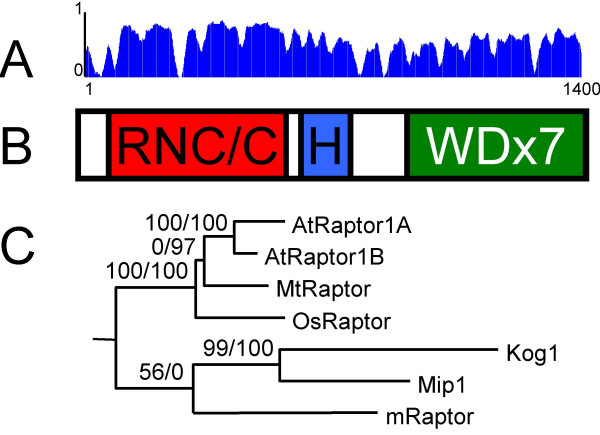
**Raptor proteins in eukaryotes are highly conserved**. (A) Similarity plot of Raptor homologues from the vascular plants Arabidopsis, *Medicago truncatula *and *Oryza sativa*, the fungus *S. pombe *(Mip1p), and mammals. The X-axis represents residue number; the Y-axis represents percent identity at that residue from 0% (0) to 100% (1). (B) Schematic diagram showing the position of the Raptor N-terminal Conserved / putative Caspase domain (RNC/C) region, HEAT repeats (H), and WD-40 repeats (WDx7) common to all Raptor proteins. (C) Phylogeny of plant, animal and fungal Raptor proteins. Bootstrap values, calculated using both parsimony (left) and maximum likelihood (right) are shown to the left of the clades they describe. The two Arabidopsis Raptor proteins, AtRaptor1A and AtRaptor1B, resolve as a single clade with 100% confidence. The alignment was generated using Megalign (DNAStar), the similarity plot was generated from this alignment using VectorNTI, and bootstrap values were calculated using PAUP*4.0b.

To determine the time of divergence between the two *AtRaptor *loci, we searched for Raptor homologues in available plant genome data using *AtRaptor1A *and *AtRaptor1B *as query sequences. Full-length Raptor loci were discovered in the available rice (*Oryza sativa *subspecies *japonica*) and alfalfa (*Medicago truncatula*) genome sequences. Using *AtRaptor1B *and partial cDNA sequence (where available) as guides, we determined putative protein sequences from these loci. Alignment of these sequences with AtRaptor1A, AtRaptor1B, mammalian Raptor, the budding yeast Raptor homologue KOG1 and the fission yeast Raptor homologue Mip1p showed that there is a striking degree of conservation among all Raptor homologues (Fig. 1A; for the complete alignment [see additional file 1]). AtRaptor1B and the *Saccharomyces cereviseae *Raptor homologue KOG1, the most divergent member included in the analysis, show 28% identity throughout their length. All plant Raptor homologues encode the Raptor N-terminal Conserved / Caspase (RNC/C) motif, HEAT repeats and WD40 motifs first identified in fission yeast Mip1 and characteristic of all Raptor proteins [25,26](Fig. 1B). From this alignment we generated a phylogeny of the Raptor homologues (Fig. 1C). The predicted AtRaptor proteins resolve to a single clade with a high degree of confidence, indicating that the duplication of the *AtRaptor *loci post-dates the divergence of *Arabidopsis *from *Medicago*, and that the loci are likely to encode functionally redundant proteins.

### Identification of knockout mutants in *AtRaptor1A *and *AtRaptor1B*

**Figure 2:**
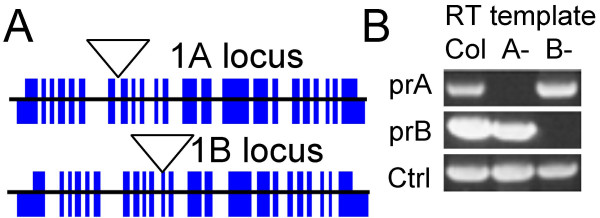
***AtRaptor *loci and insertion allele characterization**. (A) *AtRaptor1A *and *AtRaptor1B *loci. Genomic sequence is depicted as a thin central line. Thick blocks indicate exons. Coding exons span the central line; exons encoding untranslated regions are fully below the central line. The positions of the T-DNA insertions are depicted with inverted triangles. (B) Reverse-Transcribed RNA-template Polymerase Chain Reactions (RT-PCR) on plants homozygous for both wild-type *AtRaptor *alleles (Col), the *AtRaptor1A *insertion allele (A-) or the *AtRaptor1B *insertion allele (B-), using primers spanning the *AtRaptor1A *insertion site, the *AtRaptor1B *insertion site, or control primers. Both AtRaptor insertion alleles abolish accumulation of the wild-type transcript from their locus.

To gain insight into the function of the AtRaptor proteins, we searched for insertion alleles of each locus among the publicly available sequenced T-DNA insertion lines. Insertion mutant lines SALK_043920 and SALK_078159, with disruptions to the *AtRaptor1A *and *AtRaptor1B *loci, were obtained from the Arabidopsis Biological Resource Center (USA). Lines homozygous for each insertion (referred to as *1A *-/- and *1B *-/-, respectively) were identified via polymerase chain reaction (PCR), and RNA from floral buds of insertion homozygotes was used for reverse-transcriptase-PCR (RT-PCR) to assay for accumulation of wild-type transcripts from the disrupted locus. *AtRaptor1A *transcripts could not be detected in *1A *-/- buds, but were detected in *1B *-/- buds. *AtRaptor1B *transcripts were not detected in *1B *-/- buds, but were detected in *1A *-/- buds. Both transcripts were detected in wild-type Columbia (Col) buds (Fig. 2).

### *1B*-/- seedling roots grow slowly

**Figure 3:**
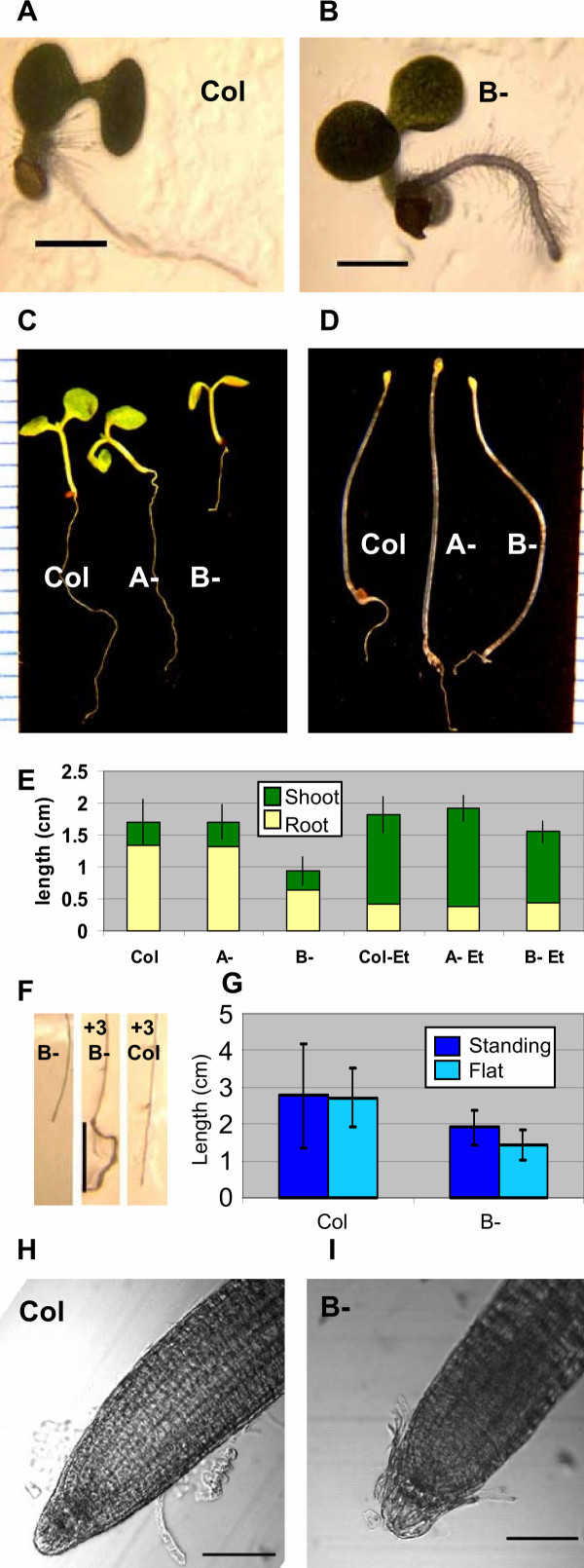
**Seedling root phenotype of *AtRaptor1B*-/- (B-) mutants**. (A), (B). Col and B- seedlings on growth medium, four days after germination. The B- root has not penetrated the medium and is thick, hairy and coiled. (C) Col, A- and B- seedlings at 8 days after germination in light. Scale bar is in mm. (D) Same genotypes and age as C, grown in the dark. (E) Measurements of populations grown as in C, D. Root length is indicated in tan; shoot length is dark green. (F) B- and Col seedlings grown on vertical plates for 12 days, and then returned to horizontal growth for three days. B- roots are thin, straight and hairless on vertical plates (compare to 3B), and revert to coiled growth only in tissue generated after being placed horizontally. (G) Quantification of results in (F). B- seedlings grown on vertical plates are intermediate in length between flat-grown B- seedlings and Col seedlings. (H), (I) Col and B- root tips, viewed under bright field microscopy. Scale bar = 100 μm. B- root tips contain all the cell types seen in Col root tips, but the overall morphology is blunt and rounded compared to Col.

As a first step toward characterizing these mutant lines, Col, *1A*-/- and *1B*-/- seedlings were germinated on culture medium. Col and *1A*-/- seedlings were phenotypically indistinguishable. However, *B*-/- seedlings showed distinct root growth defects: roots were thick, coiled and densely covered with hairs, and the roots had difficulty penetrating the medium (Fig. 3A). Plate-grown *1B *-/- seedlings were repeatedly shorter than Col or *1A*-/- seedlings. Although dark grown *1B*-/- seedlings were also shorter than Col or *1A*-/-, the difference in size was much less (Fig. 3B). While dark-grown etiolated seedlings grow primarily through the expansion and division of embryonic hypocotyl cells, light-grown seedlings grow primarily through the production of cells from the root apical meristem. Thus the difference in size between Col, *1A*-/- and *1B*-/- seedlings results primarily from a reduction in root apical meristem growth rather than a general defect in cell expansion or metabolism.

The roots of *1B*-/- seedlings grown on 90° inclined plates were morphologically wild type but shorter than Col roots. When the plates were rotated to lay flat horizontally, new growth from the root apex of *1B*-/- seedlings produced thick, coiled roots (though the roots were not densely covered in hairs), while the previously produced root tissue remained morphologically wild type (Fig. 3C). Only those sections of the root not in contact with the medium were hairy (Fig. 3A). Thus the root growth disturbance was a defect in growth into agar rather than in gravitropism, though in the absence of resistance, roots still grew more slowly than wild type.

Col and *1B*-/- root apical meristems (RAM) were examined using bright-field microscopy (Fig. 3I). *1B*-/- seedling RAMs contained all cell types present in Col RAMs but their root morphology was much more blunt; this difference appeared to be localized to the *1B*-/- zone of elongation. *1B*-/- root tips showed a tendency to shed their root caps, although shedding was sometimes observed in Col root tips as well.

### *1B*-/- shoots show developmental and morphological defects

**Figure 4:**
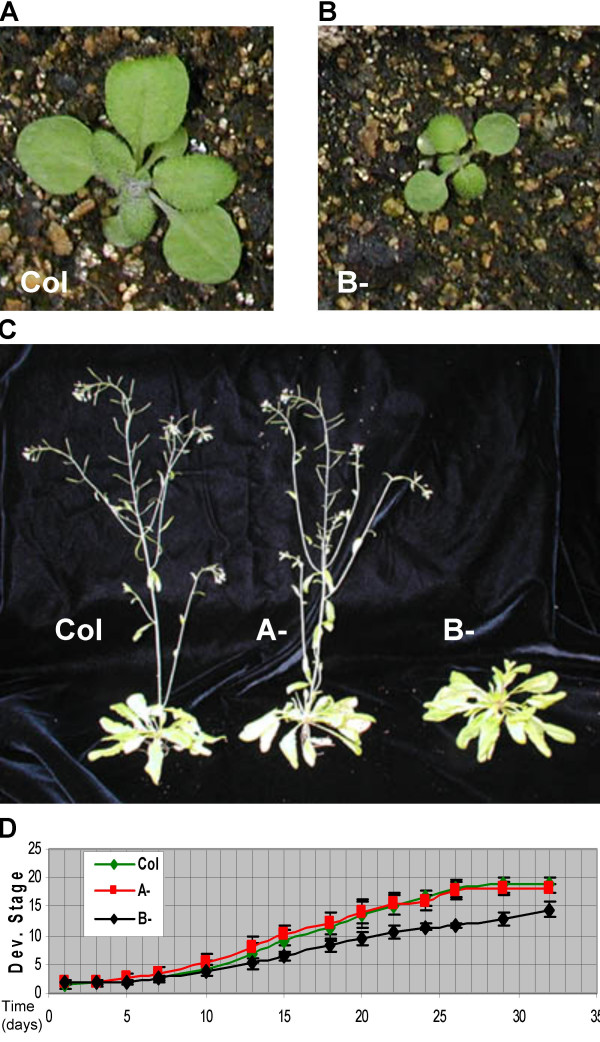
***AtRaptor1B*-/- plants grow slowly**. (A), (B) Col and B- plants at 15 days after germination on soil. (C) B- plants bolt later than Col or A-. Shown are shoots from plants 1 month after germination. (D) Growth curve of Col, A- and B- plants. The X-axis represents time after production of the first leaf. The Y-axis represents the number of rosette leaves up to 11; presence of a floral bud is 12; number of cauline leaves plus 12 is 13–16, and values above 16 are the number of shoot apices harboring flowers plus 15. B- plants show slower leaf initiation, later bolting (though at a similar rosette leaf number as Col and A-) and later flowering.

To measure developmental defects resulting from *AtRaptor *insertions, we grew Col, *1A*-/- and *1B*-/- lines on soil, and scored their rates of leaf initiation, time to floral bud initiation, rate of cauline leaf initiation, time to flowering and number of floral shoot apices. *1B*-/- plants were smaller, had a slower rate of leaf initiation, and bolted and flowered later than did Col or *1A*-/- plants (Fig. 4A–C).

**Figure 5:**
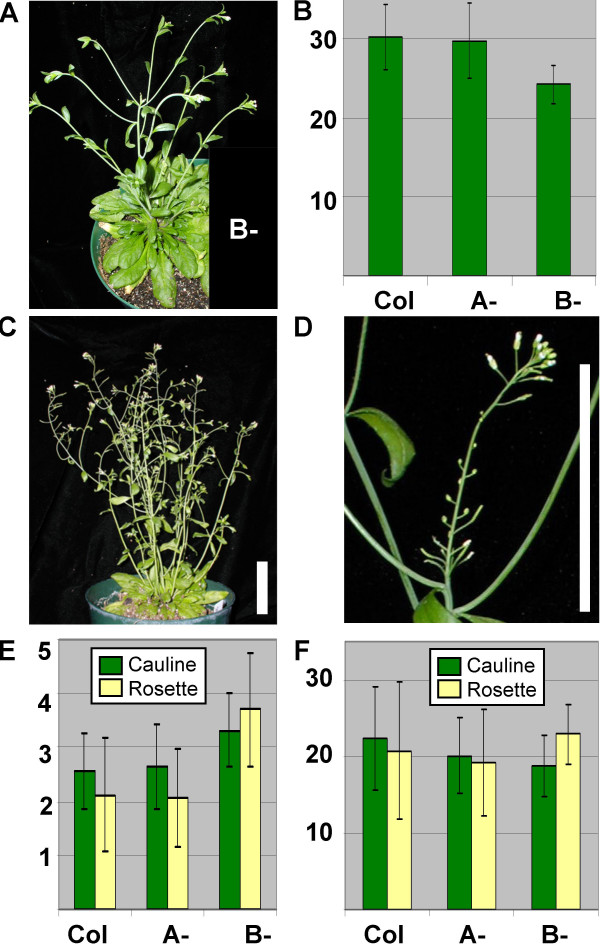
***AtRaptor1B*-/- plants show altered shoot architecture**. (A) B- plant at flowering. The primary shoot apex, center, has ceased growth and is surpassed by axillary branches. Compare to Col, A- in 3C. (B) Col, A- and B- primary shoot length. (C) Mature B- plant, showing a bushy phenotype due to decreased primary shoot growth and increased branching. (D) Shoot apex of the plant in C, magnified 4.5×. Axillary branches outgrow the primary shoot, which arrests in a whorl of sterile flowers. (E) Col, A- and B- cauline and rosette branch number. (F) Col, A- and B- cauline and rosette branch length. B- primary shoots are smaller than Col or A-, and secondary shoots initiate more frequently than Col or A- but are not significantly longer than Col or A-.

Mature *1B*-/- plants were conspicuously bushier than Col or *1A*-/- homozygotes (Fig. 5). The primary shoot apex was shorter than that of Col plants and ended prematurely in a terminal inflorescence of infertile flowers. The growth of the plant was then taken over by axillary shoots and by secondary shoots from the basal rosette.

To quantify this phenotype, Col and *1B*-/- lines were grown to maturity in 16-hr days, and shoot architecture for an individual plant was scored at the shattering of the first silique. *1B*-/- plants showed reduced shoot height, reduced primary stem length, increased axillary branch number and an increased number of secondary shoots compared to Col and *1A*-/- plants (Fig. 5B, E). *1B*-/- branch numbers, though variable, were significantly larger than those of wild type or *1A*-/-. Student's *t*-Tests of sample pairs assuming equal variance yielded P-values of 0.0027 and 0.0004 for *1B*-/- *vs*. Col and *1B*-/- *vs. 1A*-/- axillary branch number, respectively, and 0.0026 and 0.0013 for *1B*-/- *vs*. Col and *1B*-/- *vs*. *1A*-/- secondary shoot number. Axillary branch length did not differ significantly from Col values (Fig. 5F). This phenotype became more pronounced later in plant development and was more conspicuous under short days.

### Complementation of the *AtRaptor1B*-/- phenotype

To confirm that the collection of phenotypes observed in the *AtRaptor1B-/- *homozygotes was due to the mutant 1B allele, 1B-/- plants were transformed with a construct containing the *AtRaptor1B *ORF and 5' UTR and driven by the *AtRaptor1B *promoter. Some of these transformants showed wild-type transition to bolting, shoot branching and root growth (data not shown). We conclude that the *1B*-/- phenotypes described above result from homozygosity for the lesion at the *AtRaptor1B *locus.

### *AtRaptor *transcripts accumulate in dividing and expanding cell tissues

**Figure 6:**
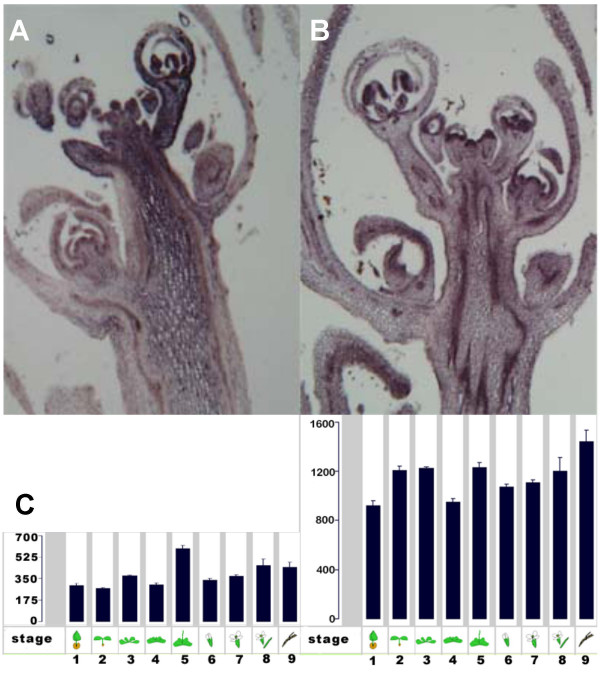
***AtRaptor *accumulation pattern**. (A) *AtRaptor *transcripts accumulate throughout the floral shoot apex, stem and differentiating floral buds. Accumulation is not confined to dividing or meristematic cells, but fades in intensity away from the apex. (B) Adjacent tissue slice, probed with actin. *AtRaptor *and actin transcript accumulation patterns differ. (C) *In silico *analysis of *AtRaptor1A *(left) and *AtRaptor1B *(right) accumulation from 1434 developmental gene chip experiments. Results are given by developmental stage (X-axis) and in terms of gene chip-normalized expression levels (Y-axis). Expression levels are shown to scale. Developmental stages are as follows: 1, 1.0–5.9 days; 2, 6.0–13.9 days; 3, 14.0–17.9 days; 4, 18.0–20.9 days; 5, 21.0–24.9 days; 6, 25.0–28.9 days; 7, 29.0–35.9 days; 8, 36.0–44.9 days; 9, 45.0–50.0 days. Analyses performed via the genevestigator website .

A combination of *in situ *RNA hybridization and *in silico *expression analysis was used to determine the RNA accumulation pattern of AtRaptor transcripts in Arabidopsis. The *AtRaptor1B *cDNA sequence was used to generate an AtRaptor probe to assay transcript accumulation in the shoot tips of wild-type plants. Since *AtRaptor1A *and *AtRaptor1B *show 80% identity through the length of their transcripts, this probe likely detected expression of both loci.*AtRaptor *transcripts were detected throughout the shoot tip, in all organs of the differentiating floral bud, and deep into the differentiated inflorescence stem (Fig. 6A). Signal intensity faded with the distance from the shoot apex. This accumulation pattern differed from that of actin (Fig. 6B), which was more prominent in dividing cells of the apex. Notably, *AtRaptor *accumulation is not restricted to the primary shoot apex.

To obtain an estimate of the relative levels of *AtRaptor1A *and *AtRaptor1B *in the signal seen in Fig. 6A, *AtRaptor1A *and *AtRaptor1B *locus IDs were used to query the Genevestigator *Arabidopsis thaliana *Microarray Database and Analysis Toolbox [27]. *AtRaptor1A *and *AtRaptor1B *accumulate in all developmental stages (Fig. 6C). However, *AtRaptor1B *accumulation levels are consistently fourfold higher than those of *AtRaptor1A*. A second analysis of accumulation by tissue rather than developmental stage produced similar results. We conclude that *AtRaptor1A *and *AtRaptor1B *show similar expression patterns.

### *1A*-/- *1B*-/- double homozygote mutant seedlings show minimal meristem growth

**Figure 7:**
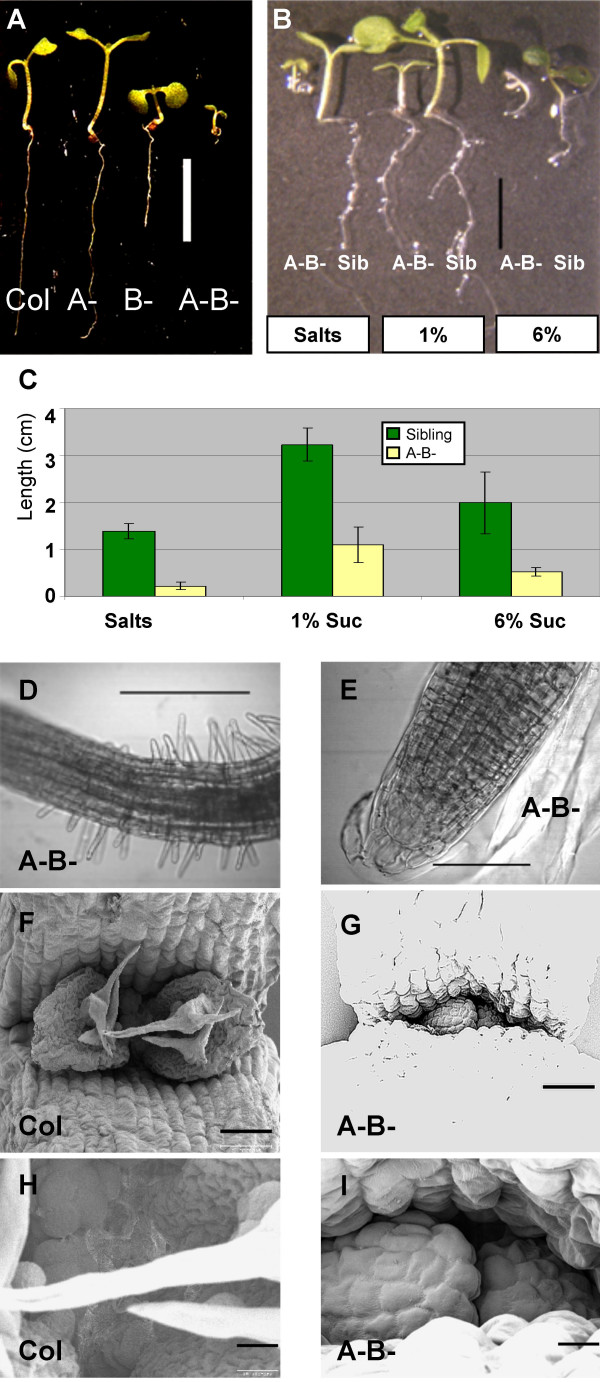
***AtRaptor1A*-/- *1B*-/- double mutants**. (A) Col, A-, B- and A-B- seedlings at seven days on growth medium with no sucrose. (B) 1A-/- 1B+/- progeny germinated on growth medium supplemented with 0%, 1% or 6% sucrose. Shown for each treatment is an A-B- seedling and an A-/- B+ sibling. A-B- seedlings on 1% sucrose show significant root growth and minimal leaf buds. Scale bar for A, B = 5 mm. (C) Quantification of results in (B). (D) A-B- root tips grown on 1% sucrose lack an epidermal cell layer. (E) A-B- roots form root hairs on 1% sucrose. Scale bar = 100 μm. Compare to 3H, I. (F, G, H, I) Scanning electron microscopy on Col, A-B- shoot apices from growth medium plates. As in (B), A-B- seedlings show minimal (SAM) activity. Primordia for leaves 1 and 2 form but do not expand significantly. Scale bar = 50 μm (F, G) or 15 μm (H, I).

The high degree of similarity between AtRaptor1A and AtRaptor1B led us to suspect that they may be at least partially functionally redundant. To eliminate all AtRaptor activity in a single line, *1A*-/- and *1B*-/- lines were crossed. No *1A*-/- *1B*-/- plants could be identified among the F_2 _of this cross on soil. Phenotypically wild-type *1A*-/- *1B*+/- plants were identified via PCR using the primer pairs described in Materials and Methods and their progeny (205 seedlings) were examined on agar plates. Of these, 165 seedlings (80%) appeared wild type (Fig. 7A). It was determined by PCR that these seedlings carried a wild-type *1B *allele (32/32 tested). The remaining seedlings (40/205; 20%) germinated but showed minimal postembryonic shoot meristem growth (Fig. 7A). It was determined by PCR that these seedlings were genetically *1A*-/- *1B*-/- (32/32 tested). This genotyping confirmed that the seedling arrest mutant phenotype co-segregated with homozygosity for the *AtRaptor1B *mutant allele in the *AtRaptor1A *mutant background. We concluded that this seedling phenotype corresponded to the *1A*-/- *1B*-/- double mutant.

We examined *1A*-/- *1B*-/- double mutant seedling roots via bright field microscopy to ascertain the extent of their post-embryonic growth defect. *1A*-/- *1B*-/- roots were conspicuously narrower than wild type. The columella, quiescent center, vasculature, pericycle, endodermis and cortex are present though all smaller than wild type; the outer layer of cells is difficult to identify. Further up the root, root hairs were clearly visible, indicating that the RAM had produced some mature root cell types (Fig. 7D, E).

To gain a better understanding of the double mutant shoot apical meristem (SAM) defect, Col and *1A*-/- *1B*-/- 7-day seedlings were fixed and coated, and their shoot apexes were observed with a scanning electron microscope. In Col seedlings, leaves 1 and 2, with trichomes, were clearly visible; primordia for leaves 3, 4 and 5 were visible under higher magnification. *1A*-/- *1B*-/- shoot apexes showed minimal SAM activity: leaves 1 and 2 were produced, but were smaller than wild type and did not separate to reveal any further leaf primordia (Fig. 7F, G, H, I).

In order to determine whether a plant hormone or signaling molecule could restore post-embryonic growth to these arrested seedlings, progeny of *1A*-/- *1B*+/- individuals were germinated on plates containing a variety of plant hormones and signaling molecules and scored for their total seedling lengths at 10 days post germination. Signaling molecules tested included the gibberellin GA3 (100 nM to 10 μM), abscisic acid (100 nM to 10 μM), the auxin 2,4-D (1 nM to 10 μM), the ethylene precursor ACC (1 μM and 10 μM), sucrose (1%) and glucose (6%). We also attempted dark treatment. None of these treatments restored wild-type SAM growth to the quarter of the progeny showing seedling arrest. *1A*-/- *1B*-/- seedlings did show a significant response to sucrose (Fig. 7B, C). Addition of 1% sucrose, which is a growth signaling factor as well as a carbon source [28], to the growth medium promoted growth in Col, *1A*-/-, *1B*-/- and *1A*-/- *1B*-/-. In sibling seedlings, the addition of sucrose elicited a twofold increase in seedling length; in the double mutants the increase was five-fold. Germination on 6% sucrose, which signals growth arrest in Col plants [28], yielded sibling plants 1–1.5× the size of seedlings grown on 1/2 Murashige & Skoog (MS) salts with no sucrose added. *1A*-/- *1B*-/- seedlings on 6% sucrose were 2.3× the length of those grown on 1/2 MS salts. A similar result was observed among *1A*-/- *1B*+/- progeny germinated in the dark on 1/2 MS salts plates, and the length of *1A*-/- *1B*-/- seedlings was increased more than 3× under these conditions. The absolute length of the double mutant seedlings was in all cases still substantially smaller that that of sibling seedlings for a given treatment, and the increase in length was largely due to hypocotyl elongation or root growth. Minimal SAM activity was observed.

## Discussion

Axillary branch growth in *Arabidopsis *and other vascular plants is governed indirectly by auxin produced at the primary shoot apex, which acts through an undetermined secondary messenger to repress axillary meristem growth [29]. The shoot morphology of *1B-/- *plants indicates a defect in primary SAM maintenance. The primary shoot is shorter than wild type and ends in a whorl of sterile flowers. The number and length of axillary shoots, both cauline and emerging from the basal rosette, is significantly increased. Notably, axillary meristems are quite viable, producing branches as long as or longer than wild type. The viability of axillary shoot apices points to a failure to maintain primary SAM activity, and a subsequent failure to repress axillary meristem activity. This defect is not shared by the axillary SAMs, which match or surpass the activity of their wild-type counterparts. A similar phenomenon is seen in *erecta *mutants [30] and may be quite common. The increased branching in *AtRaptor1B *mutant plants may result directly from reduced auxin production at the shoot apex or from a change in the auxin/cytokinin ratio. However, *1B*-/- mutants did not show defects in their ability to sense exogenous auxin (data not shown).

The endogenous *AtRaptor1A *locus cannot fully complement the disruption of *AtRaptor1B *despite the fact that their predicted proteins are 80% identical, share all conserved Raptor motifs and strongly resolve to a single clade in phylogenetic analysis. *In silico *analysis suggests that the failure of *AtRaptor1A *to mask *AtRaptor1B *lesions may be due to lower levels of *AtRaptor1A *expression globally rather than limited tissue-specific accumulation of *AtRaptor1A*. We did not assay for the complementation of *AtRaptor1B *lesions by transforming *AtRaptor1B*-/- plants with an *AtRaptor1A *construct. In contrast, *AtRaptor1B *can fully complement the loss of *AtRaptor1A *expression; indeed, a single wild-type *AtRaptor1B *allele in 1A-/- 1B+/- plants is sufficient for wild-type growth.

Our ability to isolate *AtRaptor1B *mutant homozygotes conflicts with a recent report [24]. In that report, mutants homozygous for *AtRaptor1B *disruptions were lethal at or before the first asymmetric embryonic division, which is even earlier than the lethality of *AtTOR *mutant homozygotes. We are uncertain of the reason for the discrepancy in these results. We are able to recover 1B-/- plants, but Deprost et al. [24], working with the same AtRaptor1B allele (SALK_043920), described them as embryo-lethal. One possible explanation might be a variation in growth conditions, which could lead to a higher degree of stress on developing embryos in the experiments by Deprost et al. The fact that these authors observed some embryo arrest in wild type control lines supports this possibility. Disruption of *AtRaptor1A *yielded no significant phenotype in either our growth conditions or those of the previous report [24].

Simultaneous disruption of both *AtRaptor *loci resulted in growth-arrested seedlings, which undergo normal embryonic organogenesis but are unable to maintain post-embryonic growth from their SAMs. *AtRaptor1A-/- AtRaptor1B-/- *double mutants' SAMs cease activity after the production of a few leaf primordia. We conclude that AtRaptor activity is essential for the maintenance of SAM activity, but not for SAM assembly or for the initiation of SAM activity.

Our results indicate that AtRaptor activity is not essential for embryonic organogenesis. In contrast, *AtTOR*-/- mutants arrest development early in embryogenesis [21]. The disparity between the *AtRaptor1A*-/- *1B*-/- and the *AtTOR*-/-phenotypes indicates that AtTOR activity in embryonic development does not require AtRaptor. Thus if AtTOR is acting in a complex in embryonic development, this complex does not require AtRaptor.

**Figure 8:**
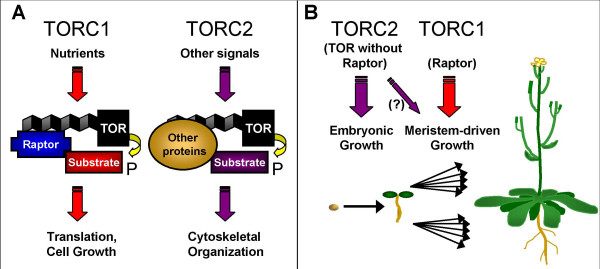
**TOR functions in two complexes in eukaryotes**. (A) TOR participates in two complexes in yeast and mammals. The first of these, TORC1, regulates cell growth in response to nutrient and hormonal signals. Raptor is integral for TORC1 activity. The second of these, TORC2, regulates cytoskeletal organization. Its activity is nutrient-independent, and Raptor is not a component of TORC2. (B) Model of TOR function in plants. Embryonic development is indicated by the single horizontal arrow from zygote to seedling; meristem-driven post-embryonic development is indicated by the arrows emanating from the seedling root and shoot apices. TOR, acting independent of Raptor in a putative complex homologous to yeast and mammalian TORC2, is essential for embryonic development. TOR via TORC2 may play a role in post-embryonic development as well; the embryonic lethal *AtTOR *knockout phenotype precludes a definitive answer on this point. Raptor activity, in a putative plant homologue of TORC1, is dispensable for embryonic development but is essential for meristem-driven post-embryonic growth.

In both yeast and mammalian cells, TOR functions in two distinct complexes (Fig. 8A). The first of these, TORC1, involves Raptor and regulates cell growth and translation in response to nutrients [14-17]. The second of these complexes, TORC2, regulates cytoskeletal organization and does not involve Raptor [13,17,20].

AtRaptor-independent AtTOR activity in the plant embryo is consistent with the existence of two AtTOR complexes in Arabidopsis: 1) a Raptor-independent complex critical for early embryogenesis (and perhaps all stages of plant development), and 2) a Raptor-dependent complex that is dispensable for embryonic development but which is necessary for post-embryonic, meristem-driven plant growth (Fig. 8B).

## Conclusion

By identifying the phenotypes resulting from partial and total disruption of AtRaptor activity, we have generated valuable tools for the study of plant TOR signaling. When viewed in the context of previous work on AtTOR [21,22], our work provides evidence for Raptor-independent TOR activity in land plant embryonic development – development that, notably, occurs in an environment made nutritionally and environmentally stable via maternal input to growth. We propose that in plants as in other eukaryotes there are two (or more) TOR complexes, only one of which involves Raptor. We further propose that the plant homologue of TORC1, involving TOR and Raptor, has been co-opted in plants from its ancestral role in nutrient sensing and cell growth to regulate the highly plastic post-embryonic growth driven by the plant shoot apical meristem.

## Methods

### Insertion lines

The mutant lines SALK_043920 and SALK_078159, in which *Agrobacterium*-mediated T-DNA insertions disrupted the *AtRaptor1A *and *AtRaptor1B *loci in the Columbia (Col) genetic background, were generated by Joseph R. Ecker and the Salk Institute Genomic Analysis Laboratory (USA) and distributed by the Arabidopsis Biological Resource Center (USA) [31]. Lines were genotyped using the following primers: 1Asm5 5'aaaaagtctcttagatgtagtttcagatg 3' and 1Asm3 5' attcagaatatacaatccaagcattagt 3' to identify the *AtRaptor1A *wild-type locus; 1Bsm5 5' ctgaccataacattctcttgtaggtaagg 3' and 1Bsm3 5' aggcctgaactctaatgaacaaactctcc 3' to identify the *AtRaptor1B *wild-type locus; and 1Bsm5 or 1Asm5 and pROK-737 (5' gggaattcactggccgtcgttttacaa 3') to identify the respective *AtRaptor *mutant alleles. Mutant lines were crossed to Col wild type plants; the *AtRaptor1B *mutant phenotype cosegregated with the 1B insertion allele in the F_2 _population.

### Growth conditions

Plants were grown in a greenhouse with supplemental light to 16 hrs, with temperatures held at 22°C days and 17°C nights. Seeds for plate-grown seedlings were surface sterilized with 20% Chlorox, washed in H_2_0, stratified for four days at 4°C and grown on 1/2× MS salts, 0.3% Phytagel under 12 hr. daylight cycles.

### RT-PCR

Buds from Col and insertion line homozygotes were snap-frozen in liquid N_2_. Total RNA was extracted from 0.3 g of tissue using TRIzol Reagent (Invitrogen) according to manufacturer's instructions. Resuspended RNA was thrice treated with DNA-free™ DNase treatment (Ambion). RNA was quantified with a spectrophotometer. cDNA was generated from 2 μg of total RNA using Omniscript Reverse Transcriptase (Qiagen) and a poly-dT_18 _primer. PCR was performed using ExTaq polymerase (Takara) in a PTC-100 thermocycler. Primer sequences were as follows: 1A+1828, 5' gctgcgtttattttggctgttattgtc 3' and 1A-2800, 5' ctaggccagccagaggagtgtgagatg 3'; 1B+1379, 5' aggccggcaaaacgatcgtaagacatt 3' and 1B-2774, 5' catcagcccagaggagccaagagg 3'. PCR cycling parameters were 95°C for 5 min followed by 35 cycles of 94°C for 30 sec, 62°C for 30 sec, and 72°C for 1 min.

### AtRaptor1B cDNA clone

EST clone RZL03b06 corresponding to the 5' end of *AtRaptor1B *was ordered from Kazusa DNA Research Institute and sequenced (Accession number AY769948). RNA ligase-mediated RACE was performed on total RNA extracted from bulk shoot tissues using a GeneRacer™ Kit (Invitrogen) according to manufacturer's instructions in order to confirm that RZL03b06 represented a full-length clone.

### Assembly of the *AtRaptor1B *complementation vector

Primers were designed to amplify the region from the end of the transcript adjacent to the *AtRaptor1B *locus to a site within the ORF, spanning a region from 1145 bases upstream of the transcript initiation site through to the sixth exon of the *AtRaptor1B *transcript. Primers used and restriction sites added are as follows: 1B-8189*Bgl*II 5' agatctgaggaaccagaagaaccc 3'; 1B+5104*Hin*dIII 5' aagcttcggcggagtaggaaaac 3'. PCR was performed using ExTaq polymerase (Takara) in a PTC-100 thermocycler on Col-0 genomic DNA with the following parameters: 96°C for 5 min, then 35 cycles of 94°C for 30 sec, 60°C for 30 sec and 72°C for 2 min, followed by a final step of 72°C for 10 minutes. *Hin*dIII *Bgl*II digested fragments of the resulting PCR products were ligated into pCambia1301.

Next, the 1301B construct was digested with *Pml*I and *Pme*I. The EST containing the *AtRaptor1B *ORF was digested with *Pme*I and *Sma*I, and the resulting fragment was ligated into 1301B to create the complementation vector 1301B:Raptor. 1301B:Raptor was transformed into *Agrobacterium *line GV3101, and then into Col or *1B*-/- plants via floral dip [32].

### Microscopy

SEM fixation was performed using standard methods [33]. Bright field microscopy was performed on a Zeiss microscope, and images were collected on a BioRad Confocal System.

### *In situ *hybridization

Digoxigenin-labelled probes were hybridized to paraffin-sectioned material using previously described methods [34].

### *In silico *analysis

Locus identifiers were submitted to the Genevestigator *Arabidopsis thaliana *microarray database and analysis toolbox [27] at , where they were assayed against 1434 developmental and tissue-specific *Arabidopsis *microarray experiment results [35-38].

## Authors' contributions

GHA and MRH formulated the experiments, analyzed the data, and wrote the manuscript, GHA obtained the data for the mutant phenotypes, and BV performed the *in situ *hybridizations.

## Supplementary Material

Additional File 1 **Alignment of Raptor homologues in plants, fungi and mammals **Additional file 1 is an image of the Raptor homologue alignment. Shown are predicted protein sequences for the plant Raptor proteins AtRaptor1A and AtRaptor1B (Arabidopsis), MtRaptor (*Medicago truncatula*), OsRaptor (*Oryza sativa*), the fungal raptor homologue Mip1 (*S. pombe*), and mammalian Raptor. Sequences were aligned in Megalign; the image was created in Genedoc. Putative sequences for AtRaptor1A and MtRaptor are based on genomic predictions rather than EST or cDNA sequence. Residues that are 100% conserved (either identical or biochemically similar) are shown as white text on black. More than 80% conservation is shown as white on dark grey, and more than 60% is shown as black on light grey. Click here for file
