# Supplementary figures and images for: The Arabidopsis AtRaptor genes are essential for post-embryonic plant growth
Source: BMC Biol. 2005 Apr 21;3:12. doi: 10.1186/1741-7007-3-12 (PMC1131892; doi:10.1186/1741-7007-3-12)

## Slide 1
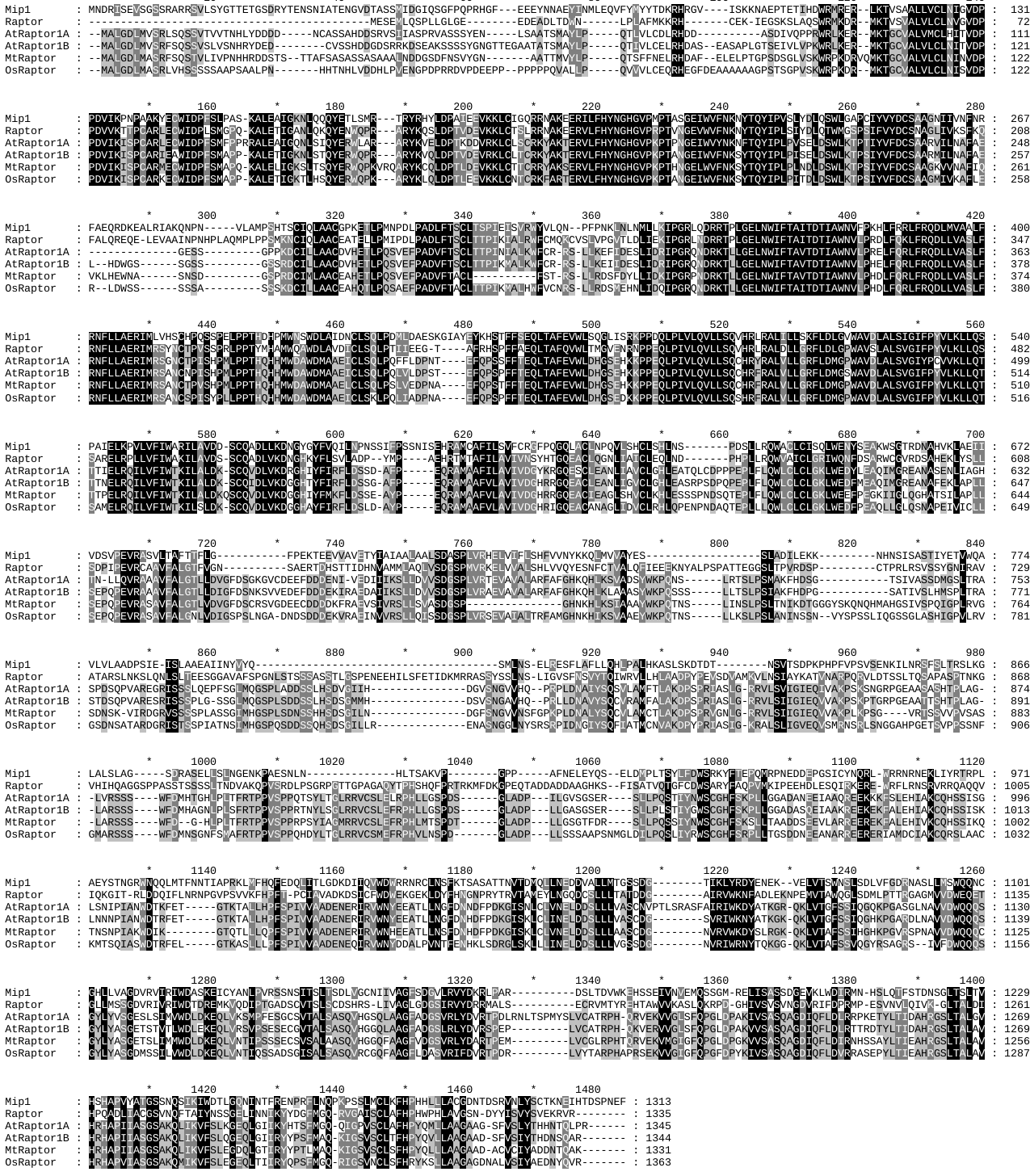

Supplement: Additional File 1 — Alignment of Raptor homologues in plants, fungi and mammals Additional file 1 is an image of the Raptor homologue alignment. Shown are predicted protein sequences for the plant Raptor proteins AtRaptor1A and AtRaptor1B (Arabidopsis), MtRaptor (Medicago truncatula), OsRaptor (Oryza sativa), the fungal raptor homologue Mip1 (S. pombe), and mammalian Raptor. Sequences were aligned in Megalign; the image was created in Genedoc. Putative sequences for AtRaptor1A and MtRaptor are based on genomic predictions rather than EST or cDNA sequence. Residues that are 100% conserved (either identical or biochemically similar) are shown as white text on black. More than 80% conservation is shown as white on dark grey, and more than 60% is shown as black on light grey. [file 1741-7007-3-12-S1.ppt]
